# Injury and Illness in World Taekwondo Junior Athletes: An Epidemiological Study

**DOI:** 10.3390/ijerph18042134

**Published:** 2021-02-22

**Authors:** Hee Seong Jeong, Sunghe Ha, Dae Hyoun Jeong, David Michael O’Sullivan, Sae Yong Lee

**Affiliations:** 1 Department of Physical Education, Yonsei University, Seoul 03722, Korea; hsj@yonsei.ac.kr; 2 International Olympic Committee Research Centre Korea, Seoul 03722, Korea; hasunghe7@gmail.com; 3 Department of Clinical Research on Rehabilitation, National Rehabilitation Center, Seoul 01022, Korea; 4 Department of Family and Community Medicine, Southern Illinois University School of Medicine, Springfield, IL 62702, USA; djeong@siumed.edu; 5 Division of Sports Science, Pusan National University, Busan 46241, Korea; 6 Institute of Convergence Science, Yonsei University, Seoul 03722, Korea

**Keywords:** adolescent, epidemiology, risk factor, injury surveillance, martial art

## Abstract

Taekwondo has been reported to be one of the most injurious sports in the summer Olympics, however, there is a dearth of data about injury profiles for junior athletes. Therefore, we aimed to identify the incidence and profiles of the injuries and illnesses that occurred during the 2018 World Taekwondo Junior Championships and recorded using an online system. Among the 889 athletes, 67 injuries and four illnesses were reported, corresponding to an overall clinical incidence of 7.5 injuries (95% confidence interval [CI]: 5.7–9.3) and 0.5 illnesses (0.1–0.9) per 100 athletes. The most frequent injuries were lower extremity injuries (*n* = 33, 3.71% of all athletes), mostly in the foot/toe (*n* = 11, 1.2% of athletes), followed by head and trunk injuries, mostly in the face (*n* = 14, 1.6% of athletes), and upper extremity injuries, mostly in the fingers (*n* = 6, 0.7% of athlete). Contusions (*n* = 37, 4.2% of athlete) were the most frequent injury type, followed by ligament ruptures/sprains and laceration. The most common injury mechanism was contact during an opponent attack (*n* = 51, 5.7% of athlete). Three mild concussions none resulted in time loss (none required hospital transfer or had prolonged recovery). The respiratory system was the most affected by illness, with pain and fever as symptoms. Environmental factors were the most common cause of illness. This study shows that 7.5 per 100 athletes (38.5/1000 athlete-exposures and 6.9/1000 min-exposures) had new or recurrent injuries, whereas 0.5 per 100 athletes experienced illness. In conclusion, the data shows male athletes reported more injuries than females and the most common cause of injury was due to contact between athletes. Contusions, ligament rupture/sprains, laceration and fractures to the lower extremities, head, and trunk were the most common injury. Knowing these injury profiles of junior taekwondo athletes can help taekwondo stakeholders, especially medical staff to prepare accordingly to ensure the safety of the athletes.

## 1. Introduction

Taekwondo is a Korean martial art. Practicing Taekwondo is well known to help improve both mental and physical health [1,2]. Taekwondo debuted as an official Olympic sport at the 2000 Sydney Summer Olympics [3]. Despite being amateurs, there are many junior Taekwondo athletes around the world who train hard and compete fiercely to win medals in the Youth Olympics and World Taekwondo competitions [4]. Even though one of the main focuses of Taekwondo as a martial art is to train the mind and body and improve health, it has a relatively high injury rate. Since the International Olympics Committee (IOC) initiated an injury surveillance system, Taekwondo has been recorded as a sport with one of the highest injury rates, consistently within the top five most injurious sports in each of the Olympic games [5,6,7]. Therefore, the governing body, World Taekwondo (WT), has been under pressure by the IOC medical committee to reduce the number and severity of injuries that occur in Olympic Taekwondo competitions [8].

There is a dearth of published research on junior injuries in Taekwondo, with most of the previous research data recorded more than 10 years ago [9,10]. Beis and colleagues [9] analyzed the injury rate of youth athletes that occurred at the 1994–1995 Greek Taekwondo Championships and reported an injury incidence of 48.21 in 1000 athlete-exposure (AEs) for male athletes and 68.97 in 1000 AEs for female athletes. Kazemi and Pieter [10] reported that junior athletes had a higher head injury rate and youth male athletes had an increased risk of sustaining a concussion as compared with adult male athletes (odds ratio [OR], 5.72; 95% confidence interval [CI], 0.67–49.14) in the Canadian National Championship. Since the previous research [9,10], there have been various equipment and rule changes [8] which may affect the injury profiles. In addition to acute injuries due to the contact nature of Taekwondo, non-impact injuries also occur owing to athletes overtraining and competing with pre-competition injuries and other chronic injuries due to automatic responses during Taekwondo competitions [11]. If these chronic injuries are not treated with the appropriate medical procedures and athletes do not give themselves sufficient recovery time, their performance can be affected drastically and irreversible injuries can occur, such as osteoarthritis [12]. Unlike senior athletes, adolescents tend not to undergo medical checkups with team physiotherapists, team medical doctors, athletic trainers, and so on; thus, they often overlook and do not understand the lasting negative impact of chronic injuries and the importance of proper medical care for injuries to prevent long-lasting degenerative changes that induce musculoskeletal dysfunctions [13]. The lack of medical support not only hinders the prevention of these chronic injuries but also prolongs the injury time and affects sporting careers [12]. Moreover, untreated injuries can hinder the normal development and growth of adolescent athleticism, causing additional medical complications at an early stage [14]. Therefore, extra care and injury prevention strategies should be implemented to protect more adolescent Taekwondo athletes from injuries.

Since 2016, WT changed the competition scoring rules by increasing the points allocated to more dynamic spinning kicks to the opponent’s head. These rule changes have been shown to increase the aggressiveness of senior athletes by incentivizing head attacks more, which could increase the probability of severe injuries [8]. In senior athletes, these new competition rules have been reported to affect the location, type, mechanism, and incidence of competition-related injuries. It can be speculated that this increase in injury trend can be extrapolated to junior athletes, as they have altered their game strategies by kicking more to the head and their subsequent training sessions have included practicing more advanced head kicking techniques such as spinning back kicks, spinning hook kicks, and roundhouse kicks [3]. To further investigate these competition rule changes and to increase the understanding of the mechanisms of injuries in Taekwondo, WT developed and initiated an online injury and illness surveillance system in 2017 to identify injuries and illnesses in athletes [15]. For accurate use of the online injury surveillance system, any medical staff who are to participate in providing medical services for WT-sanctioned competitions must attend a WT-certified training course on the procedures and processes for input into the online injury and illness surveillance system. This course includes at least one full day training on the use of a mobile on-site ultrasound device for more accurate diagnosis and has been implemented since the 2017 Muju World Taekwondo Championship (WTC) [16].

With all these new rule changes and the introduction of the protector and scoring system for junior competitions, it is paramount to have the latest injury and illness profiles to help WT and the IOC develop strategies to keep all athletes safe. Additionally, it is crucial to use the most up-to-date methods for injury evaluation to add more detail so as to understand the potential severity of the injuries. Therefore, in this study, we aimed to report the incidence and profiles of the injuries and illnesses that occurred during the 2018 World Taekwondo Junior Championships (WTJC) after the implementation of the online surveillance system by WT.

## 2. Methods

### 2.1. Participants

This prospective cohort study was conducted during the 2018 WTJC, which was held over 7 days, from 6 April 2018, to 12 April 2018, in Hammamet, Tunisia, with 889 World Taekwondo Junior athletes registered (Table 1). All the participants provided written informed consent prior to the competitions in compliance with the institutional review board of Yonsei University (IRB No. 7001988-201901-SB-245-05). We declare that the investigations were conducted in accordance with the rules of the Declaration of Helsinki of 1975, revised in 2013.

### 2.2. Study Procedure

Two methods were used to collect data. For the first method, we asked all the national team physicians to report the daily occurrence of new injuries and illnesses either on paper or by using an online database (wtfiss.com, accessed on 6 April 2018) approved by WT. For the second method, we collected injury and illness data from athletes who had been treated for any injury or illness by the organizing committee medical staff supervised by WT medical officers at the medical center in the venue and/or by specialists at designated hospitals designated by the Tunisia Taekwondo Organizing Committee. A total of five sports medical specialists (MDs) and five (certified) athletic trainers (ATs) helped in the data collection and in ensuring the safety of the participating athletes. One MD and one AT supervised each ring. For the Taekwondo-related injury profiles, we recorded the mechanism of injury and injured area. Both the MD and AT at each ring were responsible for monitoring all the injuries in each court. A portable ultrasound machine (Mindray M7 ver. 2015, Mahwah, NJ, USA) with high-frequency linear transducer (L14-6s) and convex transducer (C5-2s) was available for making a more accurate injury diagnosis. Injury data were recorded both on-site and from any designated local hospital to which the injured taekwondo athletes were transferred. The medical staff also signed the medical information release form for all the injured athletes.

### 2.3. Injury and Illness Report Forms and Definition

The online questionnaire (injury report format) was modeled based on a combination of paper and online forms used during the Rio Olympics 2016 [7] and PyeongChang Olympics 2018 [17]. The following information was recorded for each injury: the athlete’s identification number, weight division, match round, training frequency, date and time of injury, injured body part, type of injury (main symptom), and cause of injury. For the illness, absence in days, diagnosis, management or treatment, and disposition were recorded. We did not report or record any preexisting conditions and recorded only new injuries or illnesses. Any musculoskeletal complaints, concussions, or other medical conditions (injuries or illnesses) that occurred during the competition/training at the 2018 WTJC and received medical attention, regardless of the consequences with respect to absence from the competition or training, were recorded. Similarly, to other Summer and Winter Olympic epidemiological studies [5,6,7], in cases where a single incident caused multiple injury types, only the most severe diagnosis was recorded. The severity of the injury and illness was defined according to the time loss from competition/training of <1, 1–3, 4–7, or >7 days, and severity was estimated according to the nature of the injury at the time of diagnosis [17,18].

### 2.4. Statistical Analysis

All descriptive statistical analyses were performed using SPSS v24.0 (IBM Corp., Armonk, NY, USA) and Microsoft Excel (Microsoft Corp., Redmond, WA, USA). Injury and illness rates were calculated on the basis of the overall competition time recorded, with the clinical incidence recorded as the number of injuries and illnesses per 100 athletes during the 2018 WTJC [7,19]. The total number of injuries or illnesses per 1000 athlete-days was extrapolated from the 8 athlete-days we recorded [6]. The incidence rate (IR) of competition was expressed as the number of injuries per 1000 AEs (one athlete taking part in one match) and per 1000 min-exposures (MEs) [3]. The IR of the competition matches were calculated as the average injury risk for one individual athlete per 1000 AEs ([no. of injuries/no. of total AEs in the match] × 1000) [19]. The IR for MEs was calculated as the average injury risk for one individual athlete per 1000 MEs ([no. of injuries/no. of total MEs in the match] × 1000) [19]. WT-sanctioned competitions have three rounds of 2 min each, with a 1-min break [20]. The total MEs were calculated using the actual fight times, including any match that was stopped early [3]. The incidence rate ratios (IRRs) and 95% CIs were used to measure the strength of the relationships between the sexes [19].

## 3. Results

In total, 889 athletes participated in the WTJC. Among these athletes, 496 were male (55.79%) and 393 were female (44.21%). There were 869 matches, and 4832 min of competition. The weight division in which most athletes participated was 63 kg for males and 49 kg for females.

### 3.1. Overall Characteristics of the Injuries and Illnesses

Throughout the 7 days of the WTJC, we recorded 67 injuries and four illnesses, with an overall clinical incidence of 7.54 injuries per 100 athletes (95% CI, 5.73–9.34) and 0.45 illnesses per 100 athletes (95% CI, 0.01–0.89). This corresponds to 15.07 injuries per 1000 athlete-days (95% CI, 11.46–18.68) and 0.90 illnesses per 1000 athlete-days (95% CI, 0.02–1.78; Table 1).

During the competition, we recorded 38.55 injuries per 1000 AEs (95% CI, 29.32–47.78) with significantly higher rates in males (41.15/1000 AEs; 95% CI, 28.40–53.91) than in females (32.25/1000 AEs; 95% CI, 21.95–48.54). Furthermore, the total injuries per 1000 MEs were 6.93 per 1000 MEs (95% CI, 5.27–8.59), with higher rates observed in males (7.40/1000 MEs; 95% CI, 5.11–9.69) than in females (6.34/1000 MEs; 95% CI, 3.95–8.73). We observed that the male athletes were also at a higher risk of sustaining injuries during competitions than the female athletes (IRR, 1.28; 95% CI, 0.78–2.08; Table 1).

### 3.2. Location and Type of Injuries

Lower extremity injuries (*n* = 36, 4.05% of all athletes) were the most common, with the foot/toe (*n* = 11, 1.24%) being the most frequently injured area, followed by the knee (*n* = 10, 1.12%). In the head and trunk region (*n* = 20, 2.25%), the face was the most frequently injured area (*n* = 14, 1.57%), followed by the head (*n* = 4, 0.45%). Moreover, in the upper extremities (*n* = 11, 1.24%), the fingers were the most frequently injured area (*n* = 6, 0.67%), followed by the hand (*n* = 4, 0.45%) (Table 2).

Contusions/hematomas/bruises were the most common injury type (*n* = 37, 4.16%), followed by ligamentous ruptures/sprains (*n* = 7, 0.79%), laceration/abrasion (*n* = 6, 0.67%), and fractures (*n* = 5, 0.56%; Table 2). Of the three reported cases of concussions, two were mild. The athlete who sustained the third concussion was transported to the hospital, which resulted in no time-loss from the competition (Table 3).

### 3.3. Mechanisms and Intensity of Injuries

The most common injury mechanism was contact with another athlete (*n* = 51, 5.74% of athletes, 29.34/1000 AEs), followed by non-contact trauma (*n* = 9, 1.01%; Table 4). Of the injuries, 34.78% (*n* = 24) were estimated to result in no time loss from competition/training. Of 65.22% (*n* = 45) of the injuries, 31.88% (*n* = 22) were estimated to result in absence from competition/training for 1–3 days; 21.74% (*n* = 15), for 4–7 days; and 11.59% (*n* = 8), for >7 days. As an injury with a time loss of >4 days is classified as severe, this study shows that approximately 33.33% (*n* = 23) of injuries were classified as severe.

### 3.4. Injury Type-Based Injury Differences

The face was the most common site of contusion/hematoma/bruising caused primarily by contact with an opponent’s kick. The finger and knees were the most common site of ligamentous rupture/sprain, and similarly, the hand/finger and foot/toe were the most common sites of fracture (Table 5). The hand, fingers, and wrists were the most common site of fracture due to blocking an opponent’s attack. The knee and ankle were the most common sites of ligament or muscle ruptures/sprains due to both contact and non-contact events (Table 5).

### 3.5. Overall Incidence of Illness

Only four female athletes experienced an illness that affected the respiratory system. The major symptoms reported were pain (*n* = 2), fever (*n* = 1), and lethargy (*n* = 1). Environmental factors were the most common cause of the illnesses (*n* = 3), followed by infections (*n* = 1). Of the four female athletes with the illness, three received medications for mild symptoms in the onsite medical office, and one was taken to a hospital for treatment and recovered after examination. None of the cases of illness resulted in any time loss.

## 4. Discussion

In this prospective epidemiological study, we aimed to record and analyze injuries and illnesses after the implementation of the online surveillance system at the 2018 WTJC. The results are to be used to establish prevention management approaches injuries and illnesses based on the latest and most up-to-date competition rules and equipment such as the protector and scoring system, which is also used by junior athletes. Our study shows that 67 injuries and four illnesses occurred in the 889 Taekwondo athletes during the 2018 WTJC, with an overall clinical incidence of 7.54 injuries and 0.45 illnesses per 100 athletes. Among both the male and female athletes, the heavy-weight athletes had a higher injury rate than the lower-weight athletes, which is believed to be a direct result of the heavier athlete’s ability to create more powerful kicks and punches.

The overall injury incidence rate per 1000 AEs among the 2018 World Taekwondo Junior athletes (38.55; 95% CI, 29.32–47.78) was lower than those observed in the 2017 World Taekwondo athletes (77.8; 95% CI, 64.5–91.1) [21] and 2016 Korea Taekwondo Junior athletes (56.12; 95% CI, 36.97–75.27) [22]. Although the competition rules at the 2018 WTJC, 2017 WTC, and 2016 Korea Taekwondo Junior Championships (KTJC) were the same, the injury incidence rates were different (38.5/1000 AEs vs. 77.8/1000 AEs vs. 56.1/1000 AEs). The recorded overall injury incidence rate in this study was lower than those in the adult taekwondo counterparts [14]. We are not exactly sure of the reason for the lower injury incidence rate; however, on the basis of our observation during the competition, we believe that the junior athletes cannot generate as much power during their kicks as their adult counterparts could [14].

In this study, the male athletes had higher IRRs than the female athletes in the 2018 WTJC (male IRR, 1.28). Likewise, in the 2017 WTC (male IRR, 1.50), the male athletes had a higher IRR than the female athletes [21]. However, in the 2016 KTJC, the female athletes (IRR, 1.20) had a higher IRR than the male athletes [22]. A direct comparison of these 2 studies show that the risk of injury varied among competitions owing to the different rules and equipment changes [8]. In these two junior championships [21,22], the most common injury sites were the lower extremities (foot injuries due to blocking or hitting the opponent), followed by the head and trunk (face injury due to a kick to the face) and upper extremities (finger injuries due to incorrect blocking). Moreover, the most common types of injury were contusion/hematoma/bruises, followed by ligament rupture/sprain, laceration, and fracture. For medical staff attending competition, it is noteworthy to know these injury profiles (explained above) as it helpful for the staff to know what types of injuries to expect so that they can prepare accordingly.

The results of this study are similar to those of previous studies [13,22,23], showing similar injury incidence rates, injury mechanisms (mainly caused by contact with other athletes, followed by non-contact injuries), and injury locations. In addition, the mechanisms of the injuries that occurred at the national taekwondo events such as the Canadian National Championships, Thailand National Championships, and Greek National Championships were similar, with most injuries occurring owing to contact with the opponent [9,10,24]. One of the innovative factors of this research that is not available from previous junior taekwondo competition epidemiological studies is that we predicted time loss by estimating the severity of the athlete’s injury using a portable ultra-sound diagnostics device. Of all the injuries, 65% resulted in a time loss of >1–3 days and 33% resulted in a time loss of >4 days from the onset of injuries such as ligament strains and finger fractures. Therefore, coaches and athletes must include more injury prevention strategies in their training programs, such as blocking with closed fists and strengthening the joint proprioception by additional balance training methods.

This study shows that the most common injury types were contusions/hematomas/bruises to the face and lower leg (foot and toe areas). Some of the more severe injuries were ligament ruptures/sprains and finger/hand fractures, which were all caused by contact with an opponent. Logically, to prevent these types of injuries, two main preventative strategies must be used, one involving blocking and avoidance techniques and the other involving the use of more-shock-absorbent protective equipment. As the fingers tend to incur fractures while blocking, we recommend wearing protective gloves to help athletes maintain a fist position that would prevent the exposure of individual fingers to impacts [3,25,26]. As for forearm pads, shin protectors, and protective/scoring foot socks, these must have additional padding to help reduce the impact absorbed from an opponent’s attack [27,28].

In this study, a total of three sports concussions occurred (in two males and one female). Fortunately, among these cases none of the concussed athletes required transfer to a hospital or had prolonged recovery period. However, for their safety the WT has the rule that any athlete with a diagnosed concussion cannot compete at least for the next 30 days. In the 2017 WTC, five mild concussion cases occurred [21], whereas no such cases occurred in the 2016 KTJC [22]. The reason for the significant difference in the number of concussion cases according to competition year may be the changes in Taekwondo rules and the development of protective equipment [14,29,30]. Therefore, despite Taekwondo being a combat sports, it is possible that the number of injuries such as concussions in competitions can be affected by the preventative efforts of athletes, coaches, sports scientists, medical staff, and administrators based on the IOC recommendations implemented by WT [14]. To continuously prevent injuries and improve the performance of Taekwondo players, it is recommended that the TRIPP injury prevention platform proposed by Dr. Carolline Finch should be well established at all competitions and sanctioned events [31].

At the 2018 WTJC, the incidence rate of illnesses was relatively low as compared with that of injuries. The overall percentage of athletes with illnesses at the 2018 WTJC (0.4%) was lower than those at the 2017 WT Championships (3%), Rio Olympics in 2016 (5%), and London Olympics in 2012 (7%) [6,7,21]. In this study, the illnesses occurred only in the female athletes, and all four cases were confirmed to have pain, fever, and lethargy due to environmental factors and respiratory system infections. Compared with previous studies, other previous competition events had similar overall illness diagnosis, symptoms, and cause [7,13]. We believe that these illnesses are due to a plethora of issues such the requirement of athletes to fly long distances, new environments, new climate, and food intake. The stress on the body may also be magnified by the stress from competing in high-level competitions and the severe weight control athletes undergo to rapidly lose body weight, especially through the loss of essential fluids, so that they can compete at lower-weight divisions. After the rapid loss of body weight, athletes tend to load carbohydrates by eating foods with excessively high calories to prepare for the competition day [32].

### Limitations and Future Recommendations

A limitation of this study is that it was only performed at one competition and not during the training in preparation for the competition. In addition, not all the injuries may have been reported owing to the tendency of athletes to not report all their injuries and illnesses to unknown on-site medical staff at the competition. Also, a few athletes and coaches had a hard time communicating with on-site medical staff or WT medical officers due to language barriers despite onsite simultaneous interpreters. Future prospective epidemiological studies should continue to sample more data at all available competitions to make more-accurate injury profiles to help improve and develop safety and injury prevention protocols [33,34]. Injury information obtained by continually analyzing and predicting injury risk factors and applying innovative injury monitoring systems will continue to be useful tools for research to make Taekwondo safer for participants, while promoting participation for improving health [14,31,35]. In addition, as can be seen from this study and previous studies, since the characteristics of injuries and illnesses are different according to gender and weight class of adolescents, it is considered necessary to analyze the risk factors of injury and illness by further subdividing them in future studies [36,37].

## 5. Conclusions

This study shows there were more injuries were reported for the male athletes, whereas more illnesses were reported for the female athletes. Most injuries were caused by contact between athletes that caused contusions, ligament rupture/sprains, lacerations, and fractures to the lower extremities, head, and trunk. Future epidemiological studies should apply this online injury monitoring system with MDs and ATs at domestic and international Taekwondo competitions to help maintain the most up-to-date knowledge regarding injuries and illnesses not only at competitions but also during trainings. On the basis of the best available data, coaches, medical staff, and administrators will be able to develop effective safety measures to prevent injuries and illnesses.

## Figures and Tables

**Table 1:** Injury and illness characteristics of the Taekwondo athletes participating in the study.

Variable	Male	Female	Total
No. of athletes	496	393	889
Athlete-days	2480	1965	4445
Athlete-exposures	972	766	1738
Minute-exposures	5406	4258	9664
Number of injuries in competition	40	27	67
Injury clinical incidence/100 athletes ^a^	8.06 (5.57–10.56)	6.87 (4.28–9.46)	7.54 (5.73–9.34)
Injury incidence rate/1000 athlete-days ^a^	16.13 (11.13–21.13)	13.74 (8.56–18.92)	15.07 (11.46–18.68)
Injury incidence rate/1000 AEs ^a^	41.15 (28.40–53.91)	32.25 (21.95–48.54)	38.55 (29.32–47.78)
Injury incidence rate/1000 MEs ^a^	7.40 (5.11–9.69)	6.34 (3.95–8.73)	6.93 (5.27–8.59)
Injury IRR ^a^	1.28 (0.78–2.08)	0.78 (0.48–1.28)	–
Number of illnesses in competition	0	4	4
Illness clinical incidence/100 athletes ^a^	0.0	1.02 (0.02–2.02)	0.45 (0.01–0.89)
Illness incidence rate/1000 athlete-days ^a^	0.0	2.04 (0.04–4.03)	0.90 (0.02–1.78)

Note: ^a^ 95% confidence interval. AEs: athlete-exposures, MEs: minute-exposures, IRR: incidence rate ratios.

**Table 2:** Locations of the injuries that occurred at the 2018 WTJC (World Taekwondo Junior Championships).

Variable	Number of Injuries (% of Athletes)		Total
	Male	Female	
Head and trunk	13 (2.62)	7 (1.78)	20 (2.25)
Face (incl. eye, ear, and nose)	9 (1.81)	5 (1.27)	14 (1.57)
Head	3 (0.60)	1 (0.25)	4 (0.45)
Neck/cervical spine	0	1 (0.25)	1 (0.11)
Lumbar spine/lower back	1 (0.20)	0	1 (0.11)
Upper extremity	6 (1.21)	5 (1.27)	11 (1.24)
Finger	4 (0.81)	2 (0.51)	6 (0.67)
Hand	2 (0.40)	2 (0.51)	4 (0.45)
Shoulder/clavicle	0	1 (0.25)	1 (0.11)
Lower extremity	21 (4.23)	15 (3.82)	36 (4.05)
Foot/toe	8 (1.61)	3 (0.76)	11 (1.24)
Knee	4 (0.81)	6 (1.53)	10 (1.12)
Lower leg	4 (0.81)	5 (1.27)	9 (1.01)
Ankle	4 (0.81)	1 (0.25)	5 (0.56)
Groin	1 (0.20)	0	1 (0.11)
Total	40 (6.75)	25 (6.61)	67 (6.90)

Note: Data are provided as numbers (% of athletes).

**Table 3:** Types of injury that occurred at the 2018 WTJC.

Variables	No. (% of Athletes, 1000 AEs)		Total
	Male	Female	
Contusion/hematoma/bruise	22 (4.44, 22.63)	15 (3.82, 19.58)	37 (4.16, 21.29)
Ligamentous rupture/sprain	4 (0.81, 4.12)	3 (0.76, 3.92)	7 (0.79, 4.03)
Laceration/abrasion/skin lesion	2 (0.40, 2.06)	4 (1.02, 5.22)	6 (0.67, 3.45)
Fracture	5 (1.01, 5.14)	0	5 (0.56, 2.88)
Concussion	2 (0.40, 2.06)	1 (0.25, 1.31)	3 (0.34, 1.73)
Muscle-tendon rupture/strain/tendinosis	1 (0.20, 1.03)	1 (0.25, 1.31)	2 (0.22, 1.15)
Dislocation/subluxation	1 (0.20, 1.03)	1 (0.25, 1.31)	2 (0.22, 1.15)
Other bone injuries	1 (0.20, 1.03)	1 (0.25, 1.31)	2 (0.22, 1.15)
Other	2 (0.40, 2.06)	1 (0.25, 1.31)	3 (0.34, 1.73)
Total	40 (8.06, 41.15)	27 (6.87, 35.25)	67 (7.54, 38.55)

Note: Data are provided as numbers (% of athletes, 1000 athlete-exposures [AEs]).

**Table 4:** Mechanisms of the injuries that occurred at the 2018 WTJC.

Variable	No. (% of Athletes, 1000 AEs)		Total
	Male	Female	
Contact with another athlete	30 (6.05, 30.86)	21 (5.34, 20.89)	51 (5.74, 29.34)
Non-contact trauma	5 (1.01, 5.14)	4 (1.02, 5.22)	9 (1.01, 5.18)
Overuse	3 (0.60, 3.09)	1 (0.25, 1.31)	4 (0.45, 2.30)
Recurrence of previous injury	1 (0.20, 1.03)	1 (0.25, 1.31)	2 (0.22, 1.15)
Equipment failure	1 (0.20, 1.03)	0	1 (0.11, 0.58)
Total	40 (8.06, 41.15)	27 (6.87, 32.25)	67 (7.54, 38.55)

Note: Data are provided as numbers (% of athletes, 1000 athlete-exposures [AEs]).

**Table 5:** Injured body parts according to the cause and location of the injury.

Injury Type	No. (% of Athletes)					
	Male	Injured Body Part (# Causes)	Female	Injured Body Part (# Causes)	Total	Injured Body Part (# Causes)
Contusion/ hematoma/ bruise	22(4.4)	face/head (9C), foot/toe (5C), lower leg (3C), ankle (2NC, 1C), finger (1C), knee (1C)	15 (3.8)	face/head (5C), knee (3C), lower leg (2C, 1NC), hand (2C), neck (1C), foot/toe (1C)	37 (4.2)	face/head (14C), lower leg (5C, 1NC), foot/toe (6C), knee (4C), ankle (2NC, 1C), hand (2C), neck (1C), finger (1C)
Ligamentous rupture/sprain	4 (0.8)	knee (2NC, 1C), finger (1C)	3 (0.8)	finger (2C), knee (1NC)	7 (0.8)	knee (3NC, 1C), finger (3C)
Laceration/ abrasion/ skin lesion	2 (0.4)	face/head (1C), lower leg (1O)	4 (1.0)	foot/toe (2C), lower leg (1C), ankle (1C)	6 (0.7)	foot/toe (2C), lower leg (1C,1O), face/head (1C), ankle (1C)
Fracture	5 (1.0)	hand (2C), foot/toe (2C), finger (1C)	0		5 (0.6)	hand (2C), foot/toe (2C), finger (1C)
Concussion	2 (0.4)	face/head (2C)	1 (0.3)	face/head (1C)	3 (0.3)	face/head (3C)
Muscle-tendon rupture/strain/tendinosis	1 (0.2)	ankle (1NC)	1 (0.3)	knee (1NC)	2 (3.0)	ankle (1NC), knee (1NC)
Dislocation/ subluxation	1 (0.2)	finger (1O)	1 (0.3)	shoulder (1NC)	2 (0.2)	finger (1O), shoulder (1NC)
Other bone injuries	1 (0.2)	foot (1O)	1 (0.3)	lower leg (1R)	2 (0.2)	foot (1O), lower leg (1R)
Other	2 (0.4)	lumbar (1E), groin (1R)	1 (0.3)	knee (1O)	3 (0.3)	lumbar (1E), groin (1R), knee (1O)
Total	40 (8.1)		27 (6.9)		67 (7.5)	

Note: Data are provided as numbers (% of athletes). C: contact, NC: non-contact, O: overuse, E: equipment failure, R: recurrence of previous injury.

## Data Availability

The data presented in this study are available on request to the authors.
